# Author Correction: Automated imaging and identification of proteoforms directly from ovarian cancer tissue

**DOI:** 10.1038/s41467-023-43898-5

**Published:** 2023-12-01

**Authors:** John P. McGee, Pei Su, Kenneth R. Durbin, Michael A. R. Hollas, Nicholas W. Bateman, G. Larry Maxwell, Thomas P. Conrads, Ryan T. Fellers, Rafael D. Melani, Jeannie M. Camarillo, Jared O. Kafader, Neil L. Kelleher

**Affiliations:** 1 Departments of Molecular Biosciences, Chemistry, and the Feinberg School of Medicine, Northwestern University, Evanston, IL USA; 2 Proteomics Center of Excellence, Evanston, IL USA; 3 Henry M. Jackson Foundation for the Advancement of Military Medicine, Inc, Bethesda, MD USA; 4 Department of Gynecologic Surgery and Obstetrics and the Gynecologic Cancer Center of Excellence, John P. Murtha Cancer Center, Uniformed Services University of the Health Sciences, Bethesda, MD USA; 5 Women’s Health Integrated Research Center, Inova Women’s Service Line, Inova Health System, Falls Church, VA USA; 6 Department of Biochemistry and Molecular Genetics, Feinberg School of Medicine, Northwestern University, Chicago, IL USA

**Keywords:** Proteomics, Proteomics, Proteomic analysis

Correction to: *Nature Communications* 10.1038/s41467-023-42208-3, published online 14 October 2023

The original version of the Article contained the following errors in Fig. 2: the proteoform signature ‘7125 Da’ was incorrectly labelled as ‘PSMD1’ in panels b and c; the labels ‘237 tumor pixels’ and ‘235 stroma pixels’ were incorrectly added to panel d and they have been removed in the correct version. The correct version of Fig. 2 is:
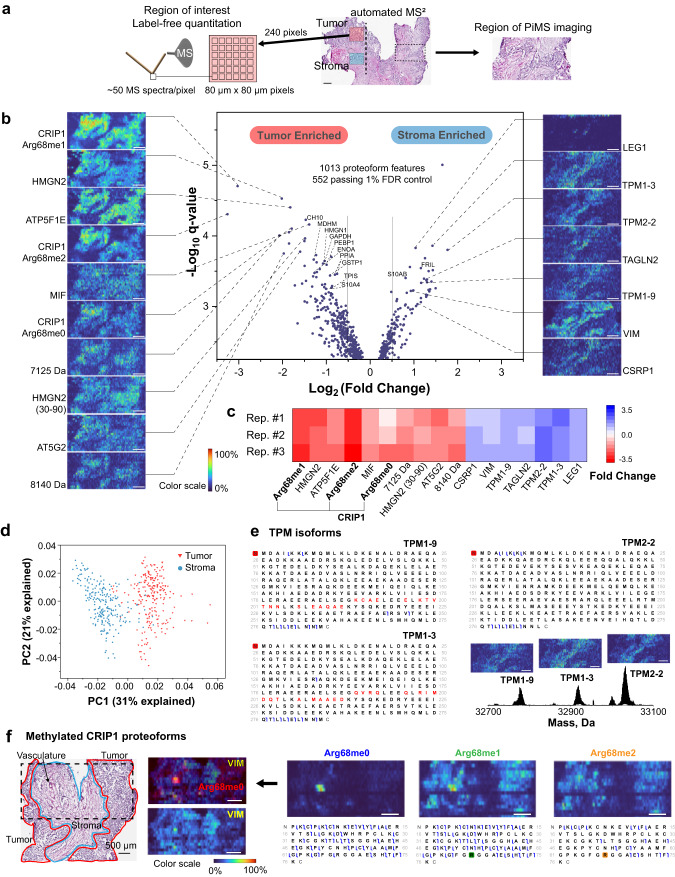


which replaces the previous incorrect version:
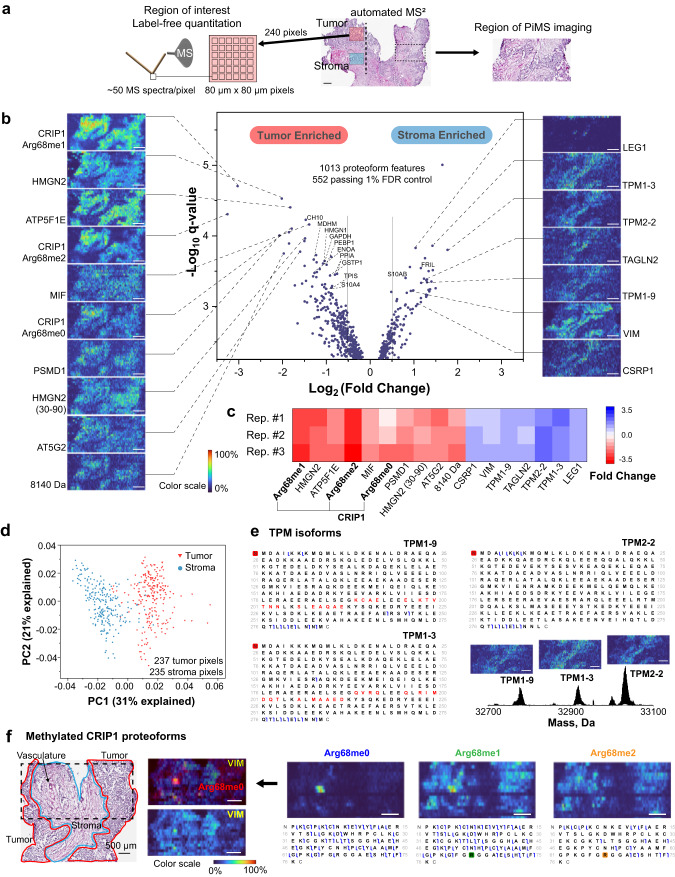


In addition, the original version of this Article contained an error in the Methods section, which incorrectly read ‘Spatial pixel allocation algorithm’. The correct version states ‘Spatial bin allocation algorithm’ in place of ‘Spatial pixel allocation algorithm’. Lastly, in the original version of this Article, the funding details for NIH HuBMAP grant UH3CA246635 (N.L.K.) were omitted in the Acknowledgements section. All these errors have been amended in both the PDF and HTML versions of the Article.

