# Supplementary material for: Automated imaging and identification of proteoforms directly from ovarian cancer tissue
Source: Nat Commun. 2023 Oct 14;14:6478. doi: 10.1038/s41467-023-42208-3 (PMC10576781; doi:10.1038/s41467-023-42208-3)
Supplement: Supplementary file 1 — Supplementary Information [file 41467_2023_42208_MOESM1_ESM.pdf]

Supplementary Information

Automated imaging and identification of proteoforms directly from ovarian cancer tissue

John P. McGee,[1+] Pei Su,[1+] Kenneth R. Durbin,[2] Michael A. R. Hollas,[2] Nicholas W. Bateman,[3,4] G. Larry Maxwell,[4,5] Thomas P. Conrads,[4,5] Ryan T. Fellers,[2] Rafael D. Melani,[1] Jeannie M. Camarillo,[1,6] Jared O. Kafader,[1,6] and Neil L. Kelleher[1,2,6]*

Affiliations

[1]Departments of Molecular Biosciences, Chemistry, and the Feinberg School of Medicine, Northwestern University, Evanston, IL, USA

[2]Proteomics Center of Excellence, Evanston, IL, USA

[3]Henry M. Jackson Foundation for the Advancement of Military Medicine, Inc, Bethesda, MD, USA

[4]Department of Gynecologic Surgery and Obstetrics and the Gynecologic Cancer Center of Excellence, John P. Murtha Cancer Center, Uniformed Services University of the Health Sciences, Bethesda, MD, USA

[5]Women's Health Integrated Research Center, Inova Women's Service Line, Inova Health System, Falls Church, VA, USA

[6]Department of Biochemistry and Molecular Genetics, Feinberg School of Medicine, Northwestern University, Chicago, IL, USA

[+]These authors contribute equally.

*Correspondence to: Neil L Kelleher, n-kelleher@northwestern.edu

Supplementary Figures

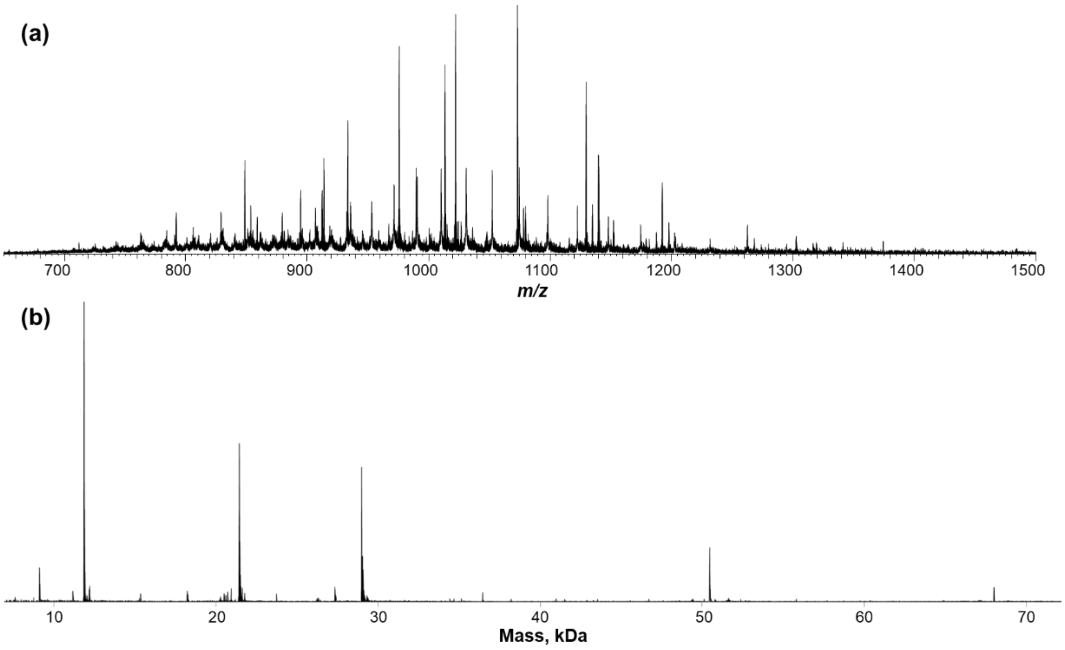

(c)

Mass	*m/z*	Charge	Component Ion Count	Co-isolated Ion Count	Component Percentage
9112.634	912.3	10	387	4	99.0%
11865.51	848.5	14	2329	0	100.0%
21444.63	1341.3	16	733	10	98.7%
28982.91	1160.3	25	346	25	93.3%
50462.94	1442.8	35	111	19	85.4%
68004.38	986.6	69	169	0	100.0%

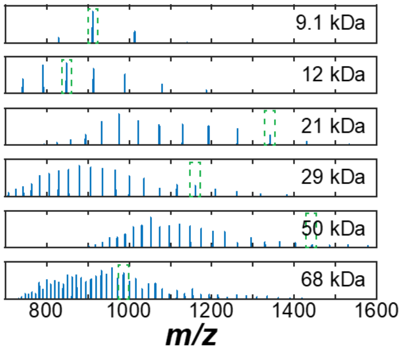

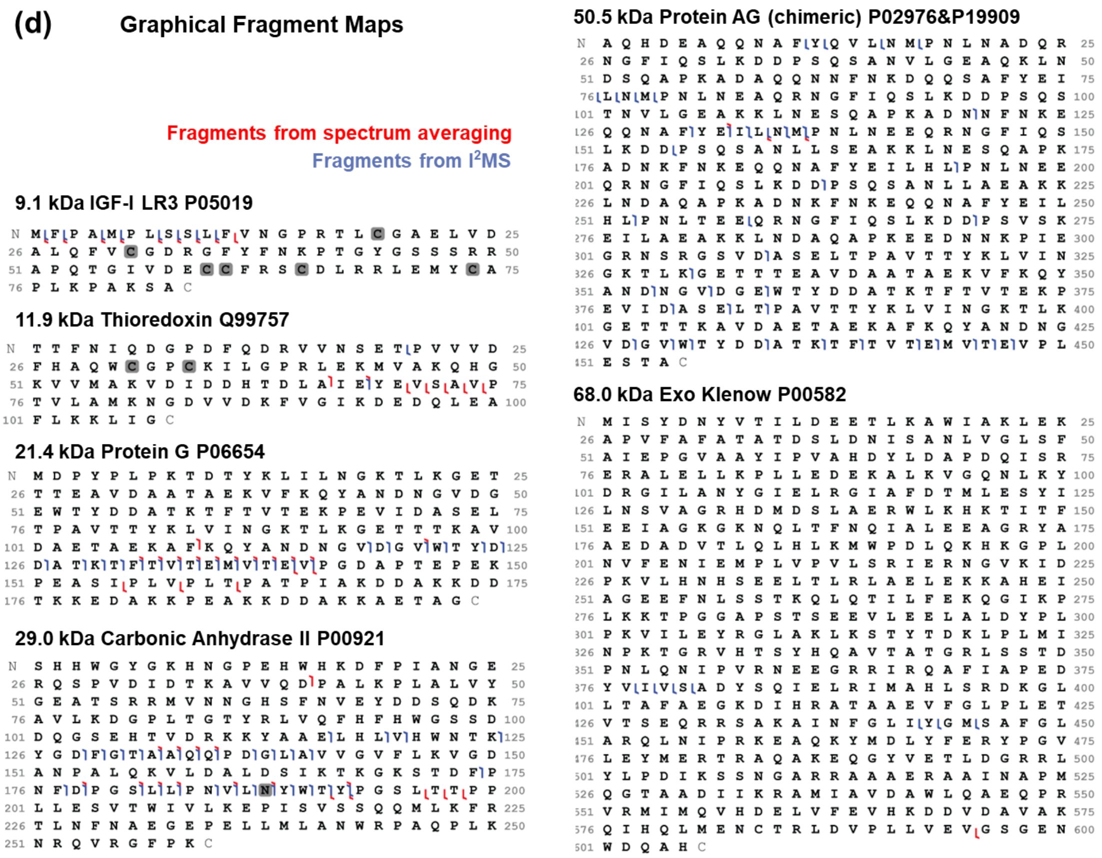

Supplementary Figure 1. (a) *m/z*- and (b) mass-domain spectra of Pierce intact protein mixture. (c) output of the algorithm for *m/z* isolation window selections for all six constituent proteins. The chart contains information for each protein: average mass, selected *m/z* isolation window, charge state, total ion count of the charge state, other species co-isolated in the selected *m/z* isolation window, and the relative abundance of the target protein in the selected *m/z* isolation window. The reconstructed *m/z* spectra of the six proteins and selected *m/z* isolation windows are shown in the right panel. (d) Graphical fragment maps of the six proteins from their corresponding targeted fragmentation experiments using the algorithm's recommendations. Red and blue labels correspond to fragments identified using averaged composite spectrum and I^{2}MS, respectively. Grey squares indicate disulfide bond locations (C) or deamidation (N).

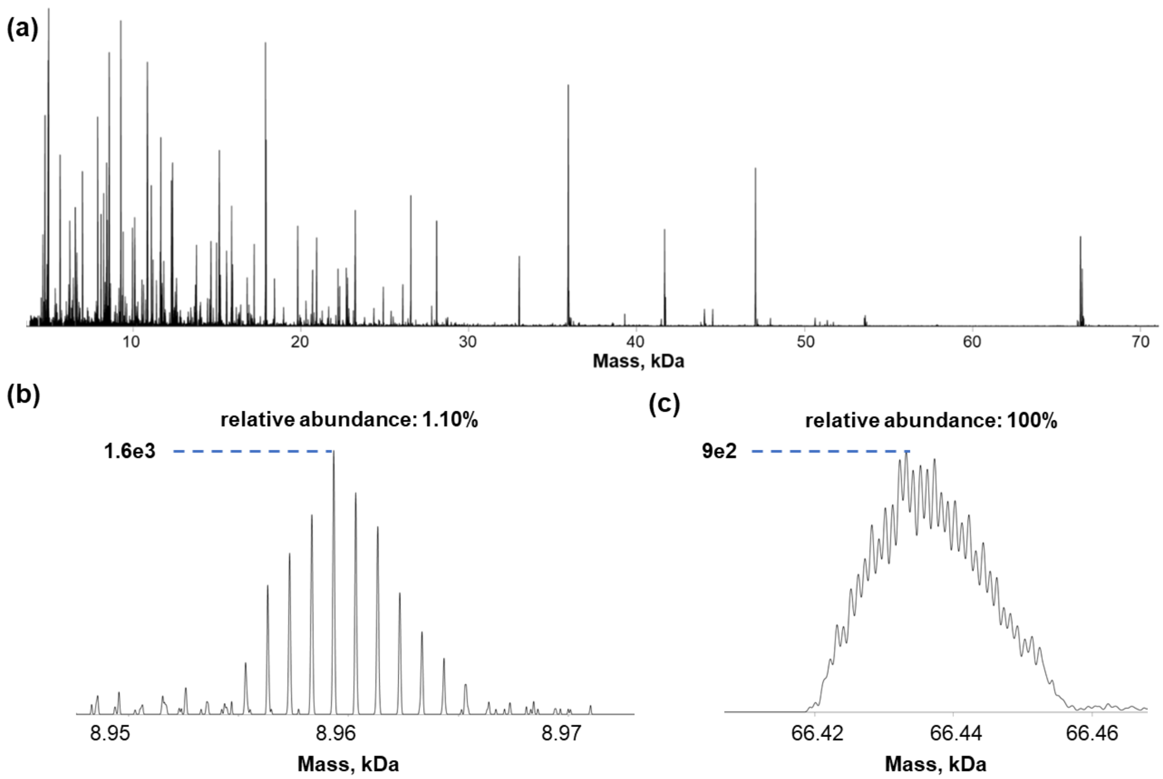

Supplementary Figure 2. (a) Full mass spectrum of the AutoPiMS survey line scan on the high-grade serous ovarian cancer tissue containing 113 proteoforms with greater than 1% relative abundance ranging from 4-67 kDa. The survey line scan data was acquired in the I^2MS mode on Q Exactive Plus instrument with a modified Orbitrap central electrode voltage of 1 kV. Zoomed in spectral range containing proteoform at 1% (b) and (c) 100% relative abundances. The dashed line indicates the absolute abundances at the level of the most abundant isotopic peak of the corresponding proteoform. The discrepancy in relative and absolute abundances is a result of the number of isotopic channels of proteoforms in drastically different mass ranges.

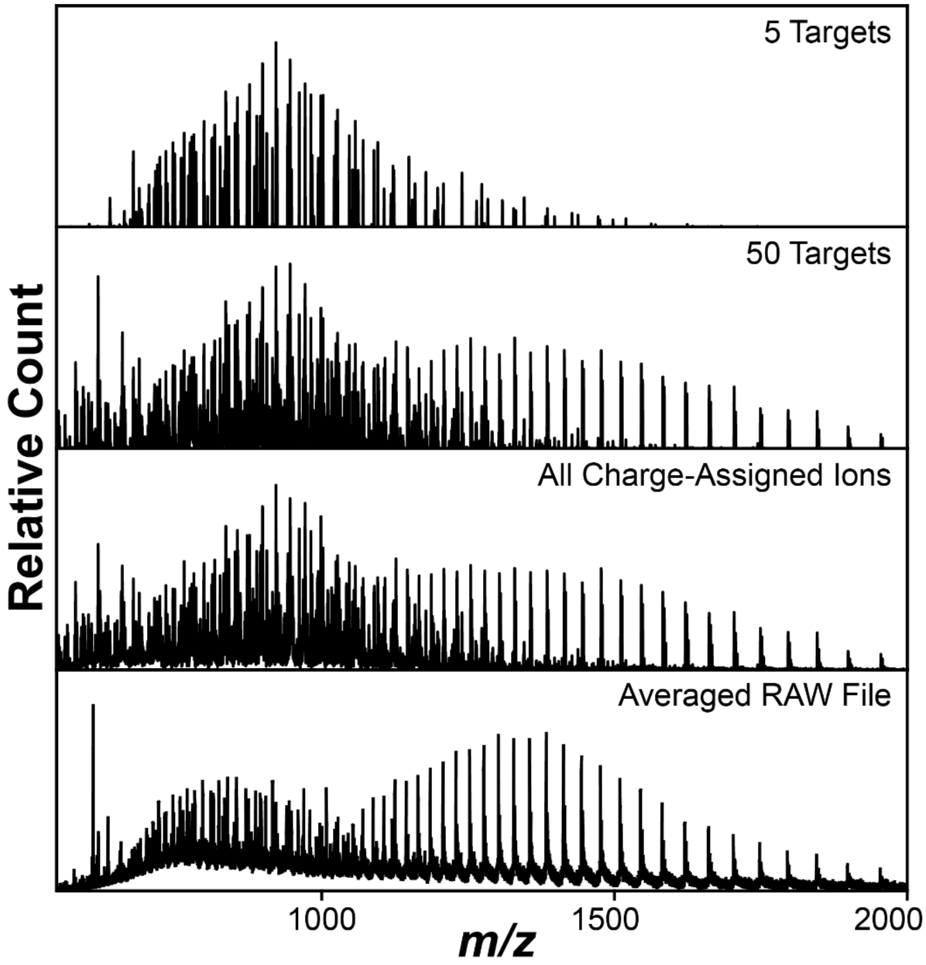

Supplementary Figure 3. Reconstruction of *m/z* spectra of the same AutoPiMS survey line scan data in **Supplementary Fig. 2** using the top 5 targets, the top 50 targets, all charge assigned ions, and the averaged spectrum from the corresponding .RAW file showing the complexity of the spectrum increases as more information is included.

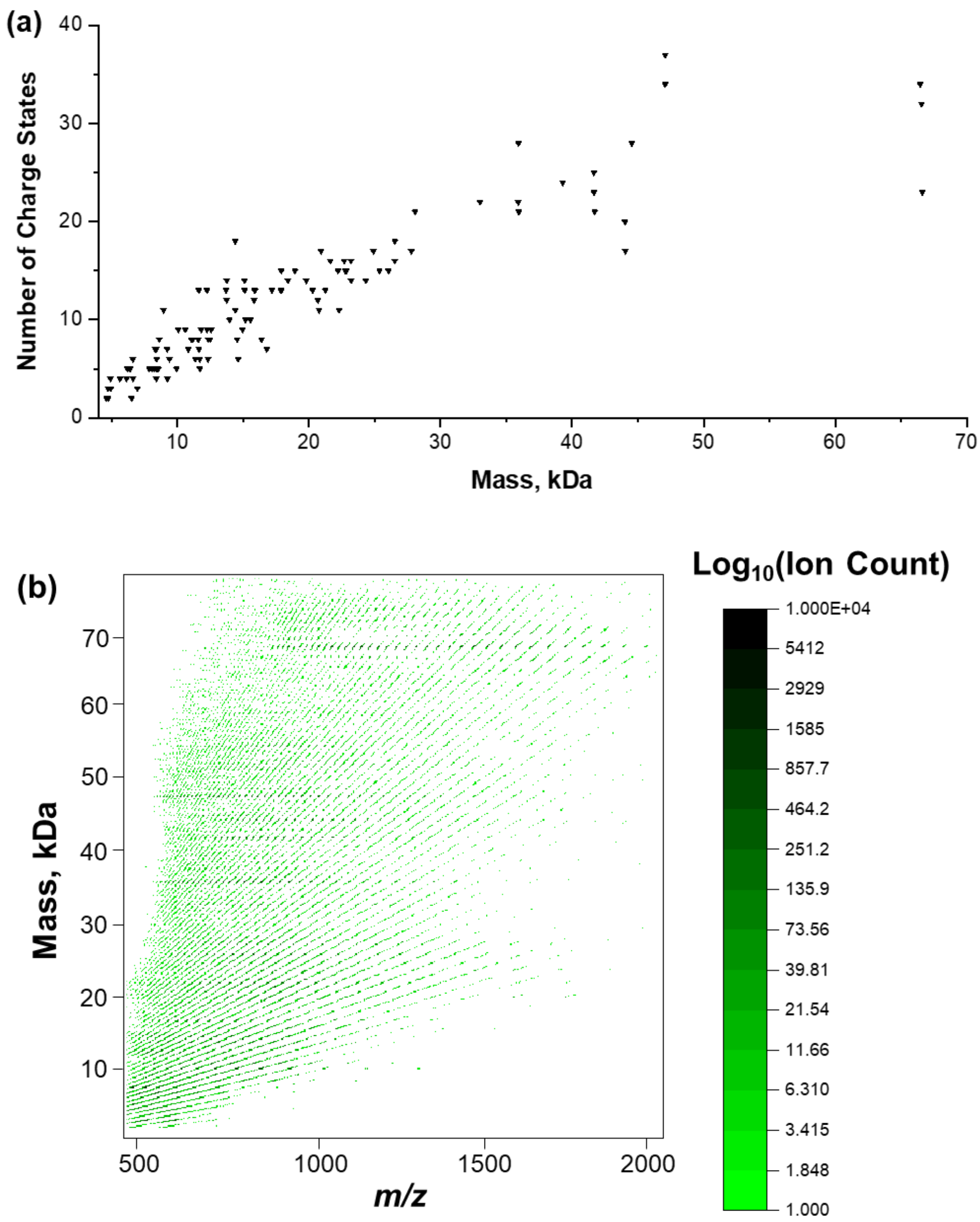

Supplementary Figure 4. (a) Number of detected charge states of the 113 proteoforms in **Supplementary Table 1** plotted against average mass; (b) A heatmap of the *m/z* and average masses of the 113 proteoforms.

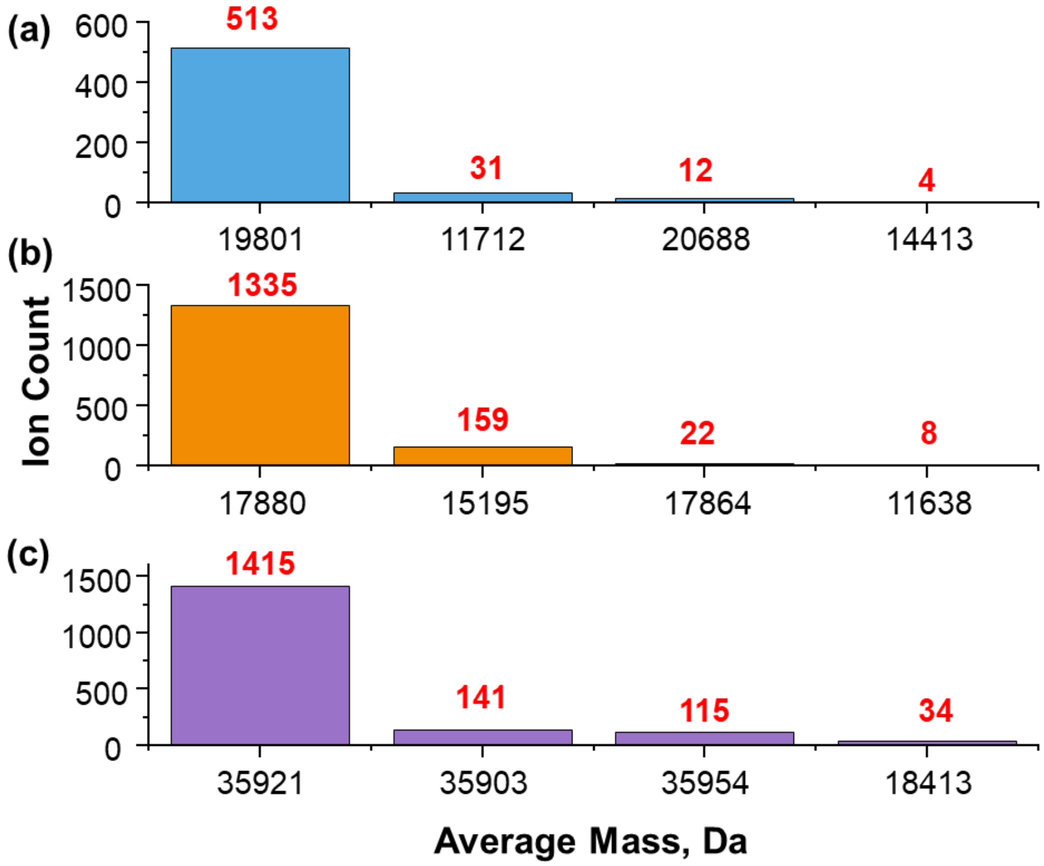

Supplementary Figure 5. Ion counts from different proteoforms present in the AutoPiMS-selected *m/z* isolation windows (0.8 *m/z*) for the proteoforms of average masses of 19801 Da (a), 17880 Da (b), and 35921 Da (c). In each panel, proteoforms are listed from high to low in ion count. The red numbers in each panel indicate the absolute ion count of that proteoform in the isolation window. As shown in this figure, the target proteoforms we intended to isolate were present with substantially higher ion count than other co-isolated proteoforms.

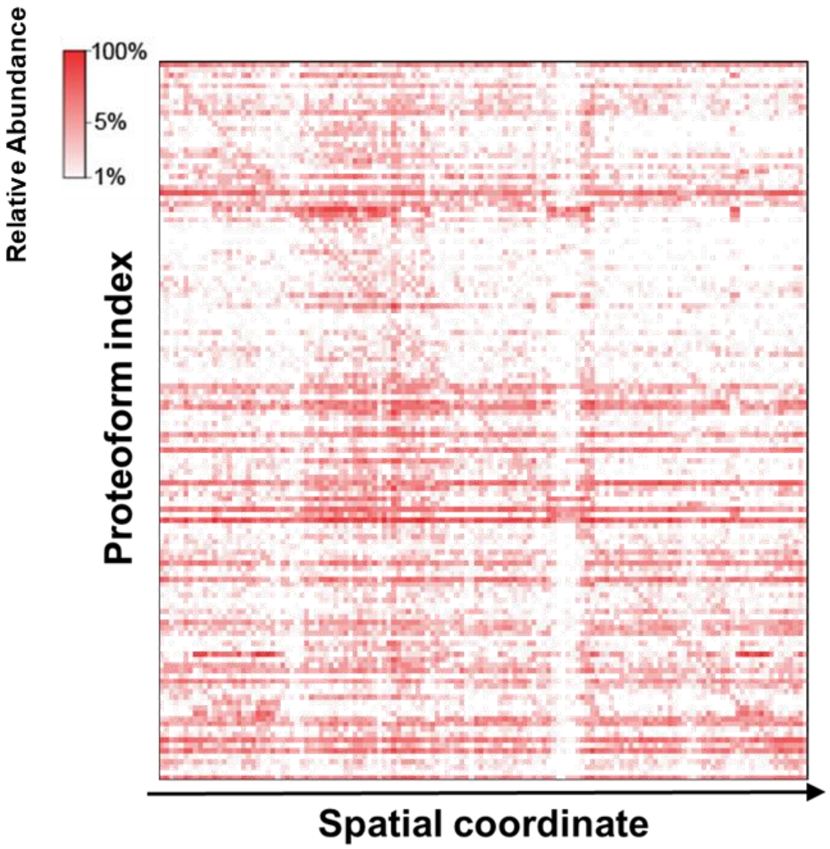

Supplementary Figure 6. A heatmap of spatial relative abundances of 79-targeted proteoforms detected in the <17 kDa range in a representative survey line scan on the HGSOC tissue described in **Fig. 1a** (**Supplementary Table 6**). Each row represents the spatial relative abundances of a proteoform. The proteoforms (*y*-axis from top to bottom) are organized according to the spatial targeting sequence (topmost and bottommost proteoforms are targeted in the leftmost and rightmost bin, respectively). The color bar on the top left shows the relative abundances for the bins. Upon automated MS^2 algorithm optimization, all the proteoform targets were assigned with a spatial bin with a relatively high abundance (diagonal bins are illuminated).

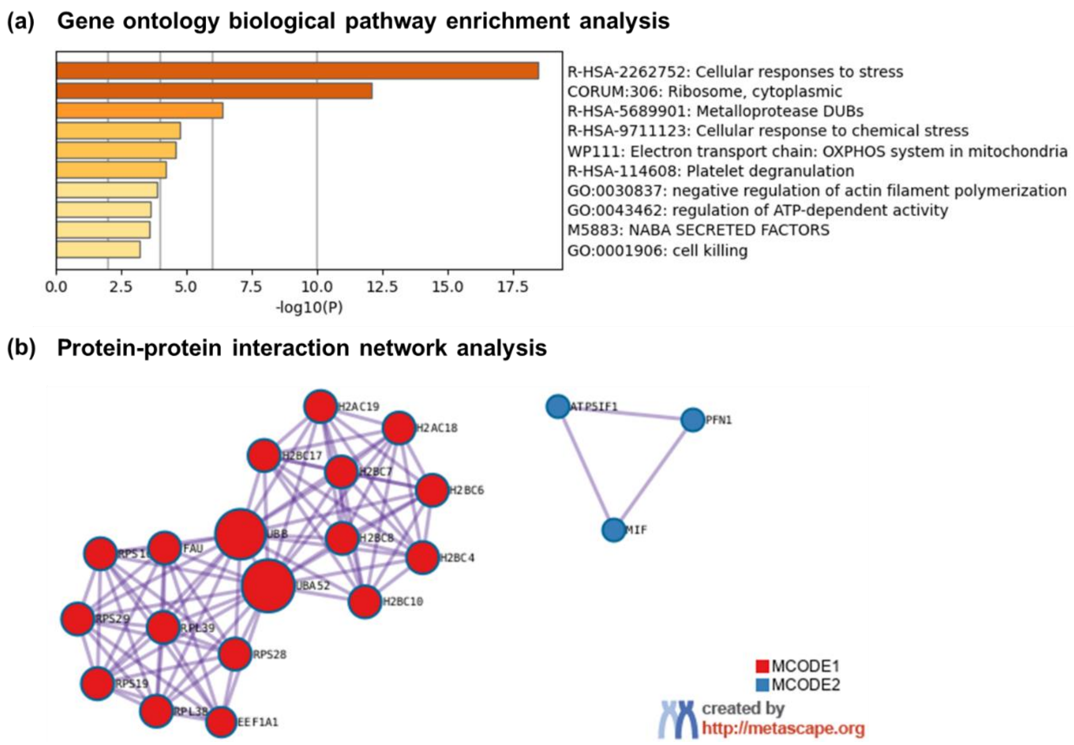

Supplementary Figure 7. GO analysis of the 73 MS^2-identified proteoforms (a), showing “Cellular responses to stress” is the most enriched pathway. Protein-protein interaction (PPI) network analysis results of the 73 proteoforms are shown in (b).

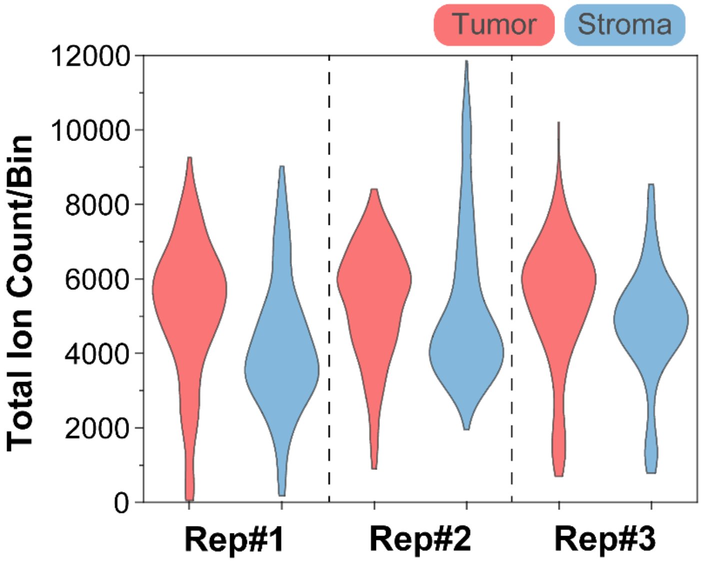

Supplementary Figure 8. Total ion count distributions of the tumor and stroma sampled regions in the region-of-interest LFQ sampling (Rep#1 corresponds to the data shown in **Fig. 2d**, and Rep#2 and #3 are technical replicates from two adjacent tissue sections).

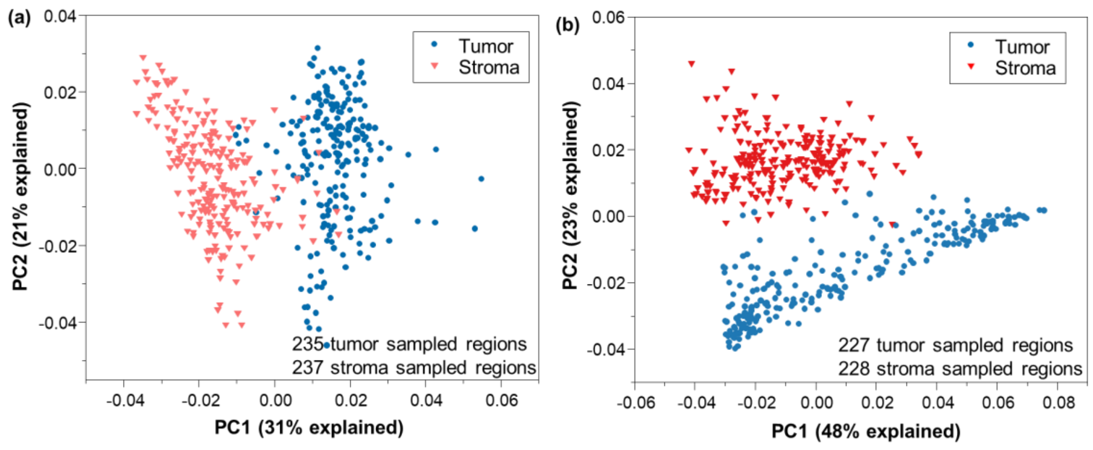

Supplementary Figure 9. Unsupervised classification of tumor and stroma sampled regions in the two technical replicates using principal component analysis in adjacent tissue sections.

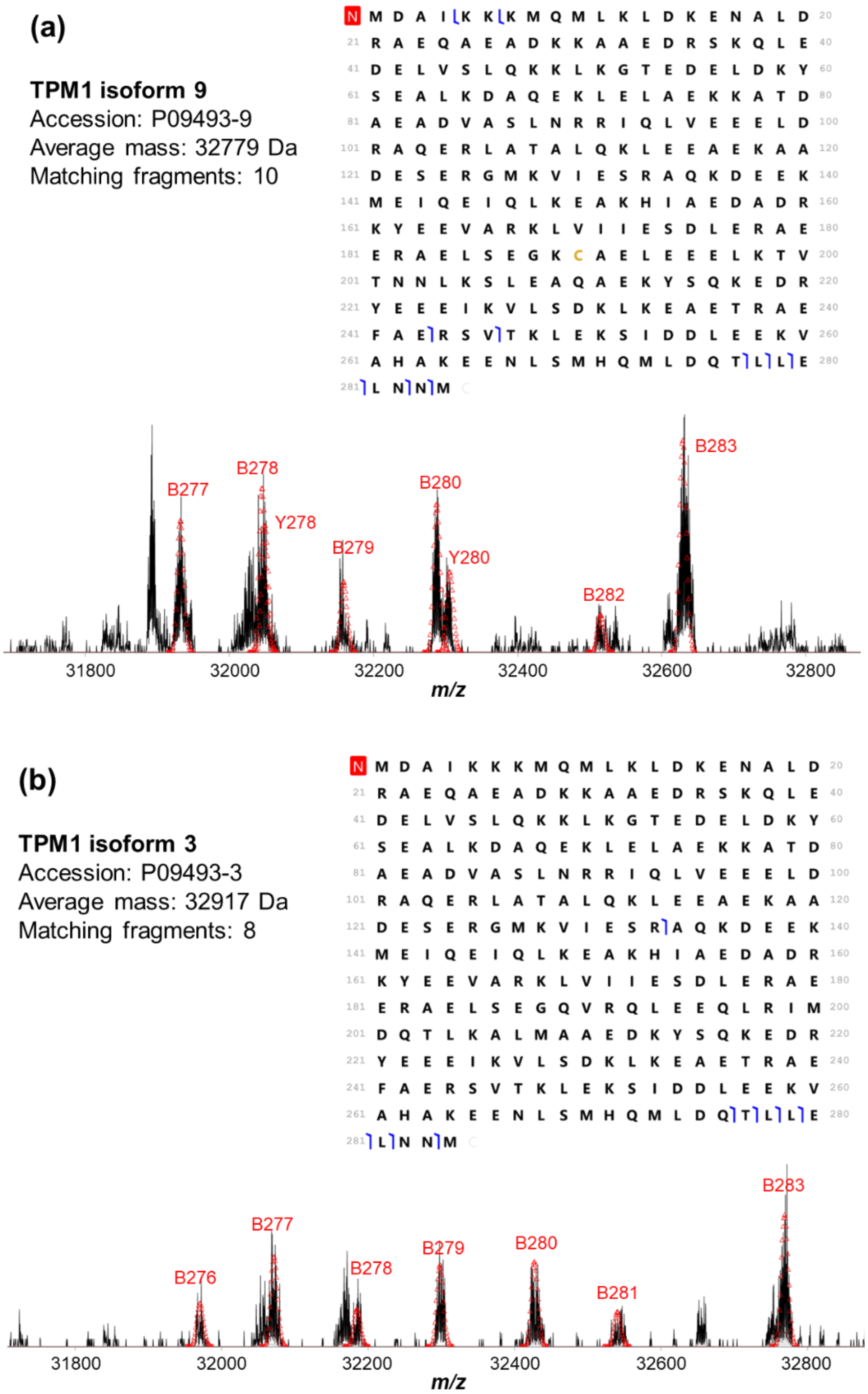

Supplementary Figure 10. Graphical fragment maps and MS^2 spectra of TPM1 isoforms shown in **Fig. 2e** (TPM1-9 and TPM1-3).

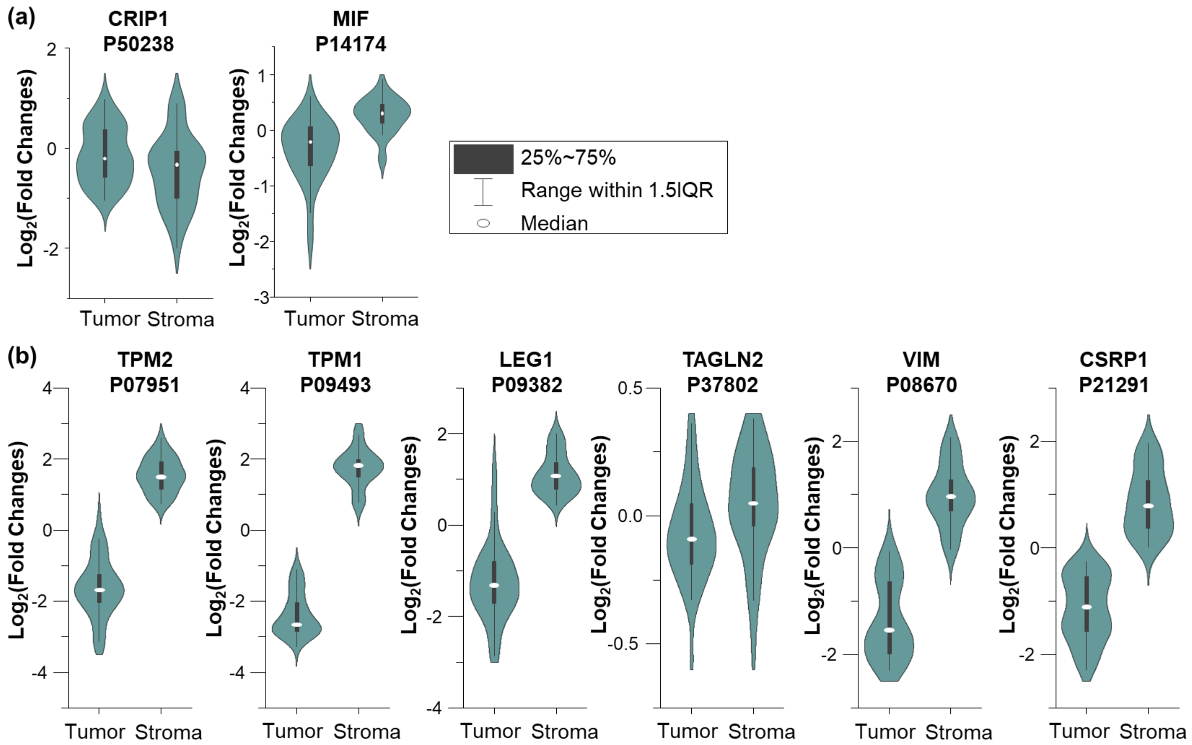

Supplementary Figure 11. Tumor- and stroma-enriched bottom-up proteomics results on HGSOC biopsies.[1] Protein abundances of the proteoform signatures discussed in **Fig. 2** that show elevated tumor or stromal detection are shown in (a) and (b), respectively.

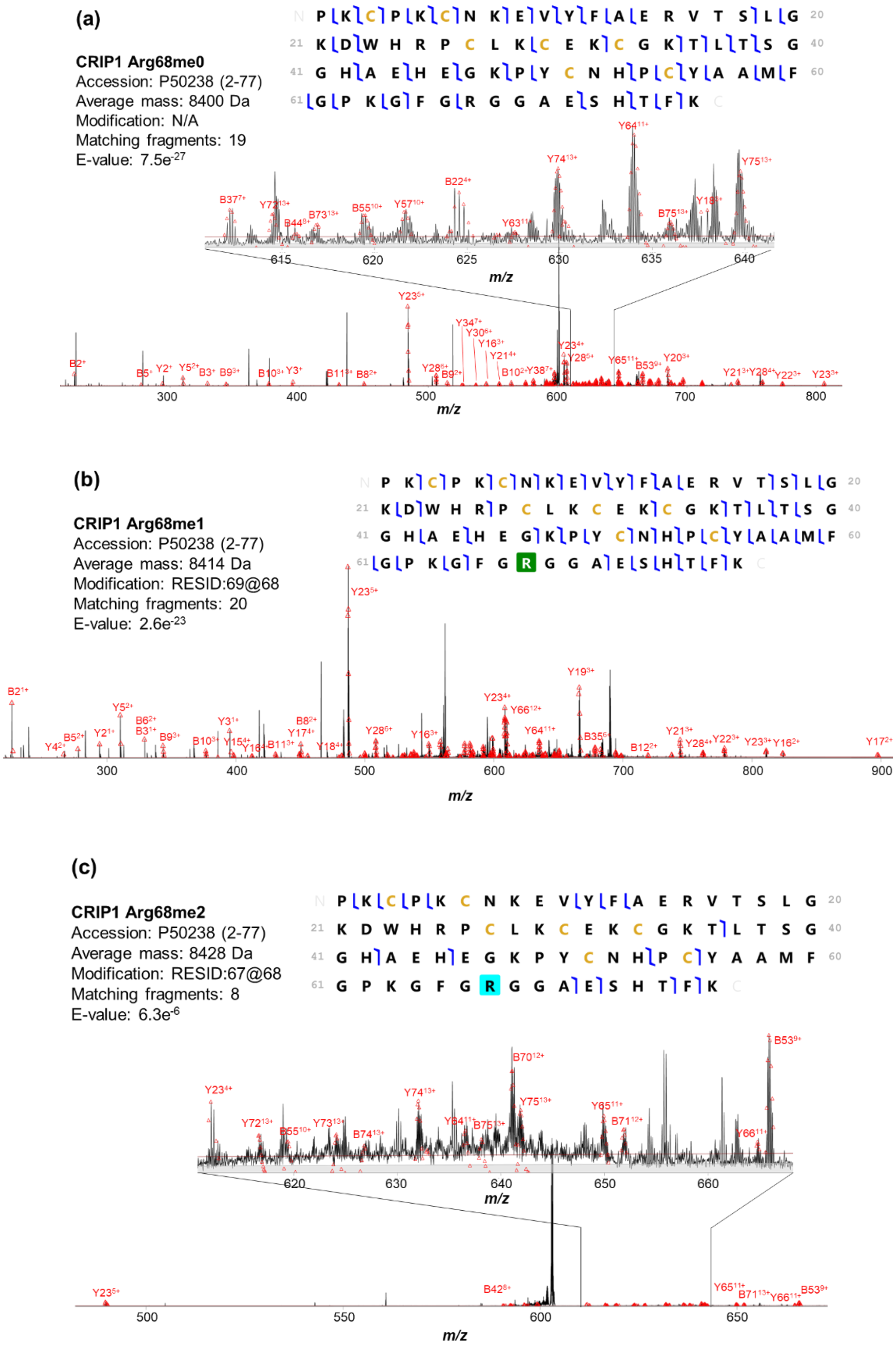

Supplementary Figure 12. Graphical fragment maps and MS^2 spectra of CRIP1 proteoforms (Arg68me0, Arg68me1, Arg68me2) shown in **Fig. 2f**.

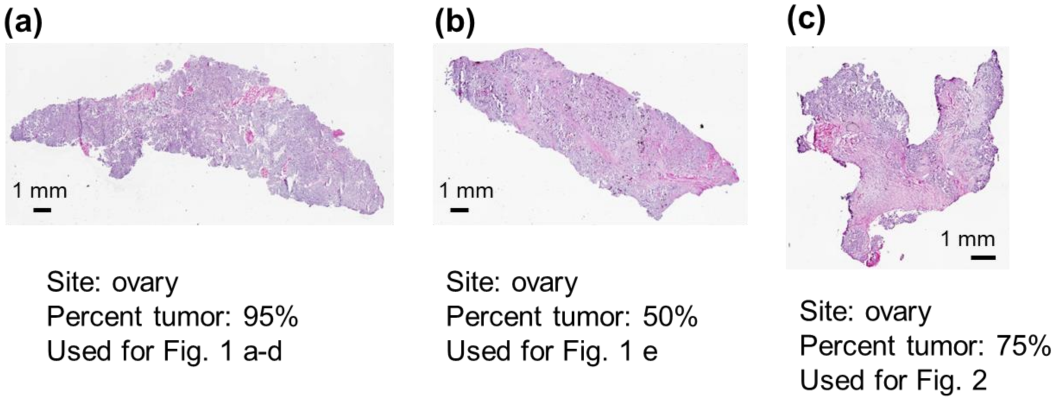

Supplementary Figure 13. Extended information of the human high-grade serous ovarian cancer tissue sections used in this study.

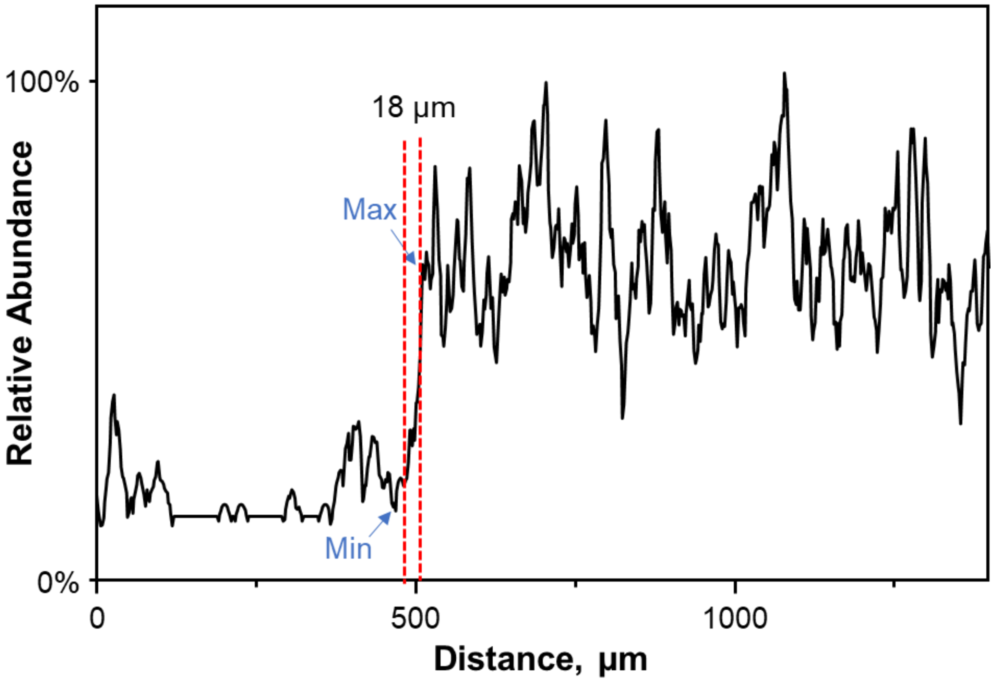

Supplementary Figure 14. Estimation of the spatial resolution of the imaging experiment. The spatial resolution is defined by the distance between 20% and 80% relative abundance of a sharp change in a spatial line profile of CRIP1 Arg68me0 in the imaged region corresponding to a boundary of the tissue.[2]

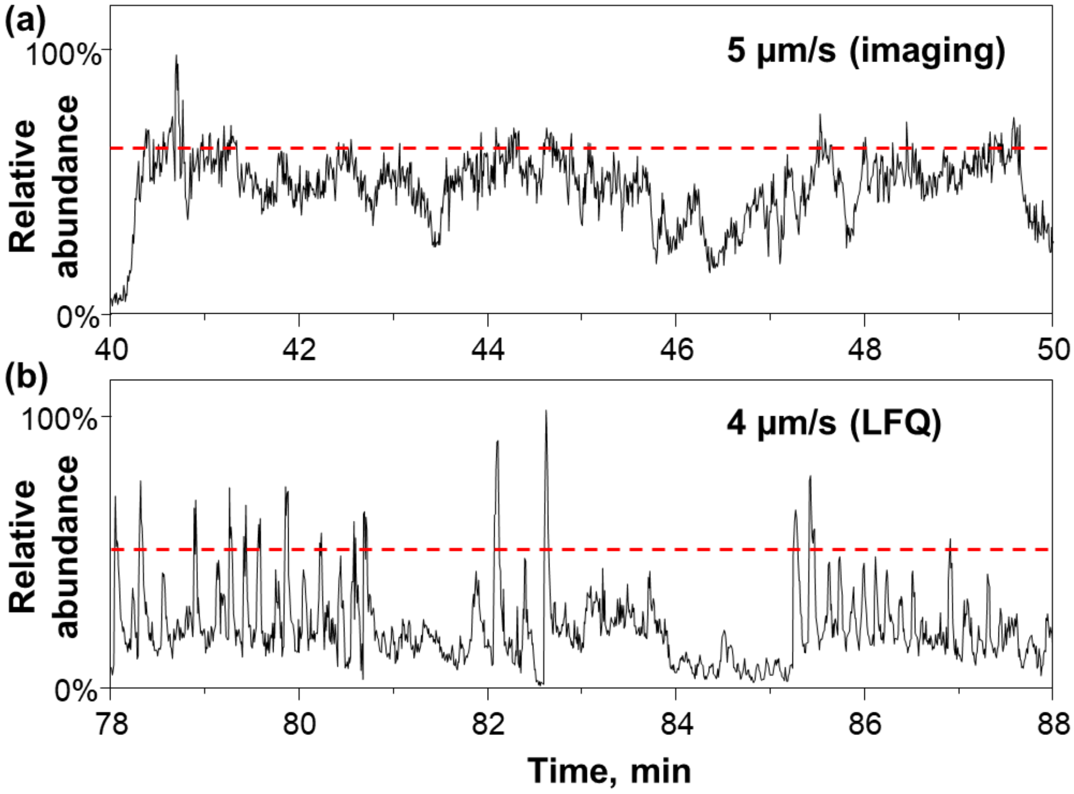

Supplementary Figure 15. Typical imaging profile at 5 μm/s (a) and LFQ profile at 4 μm/s (b). The red dashed lines indicate the same absolute total ion count level in both panels. In the LFQ profile (b), the spike features with highest ion counts (at the tip of the spike) are ~10% to 20% higher than the average profile in imaging experiment.

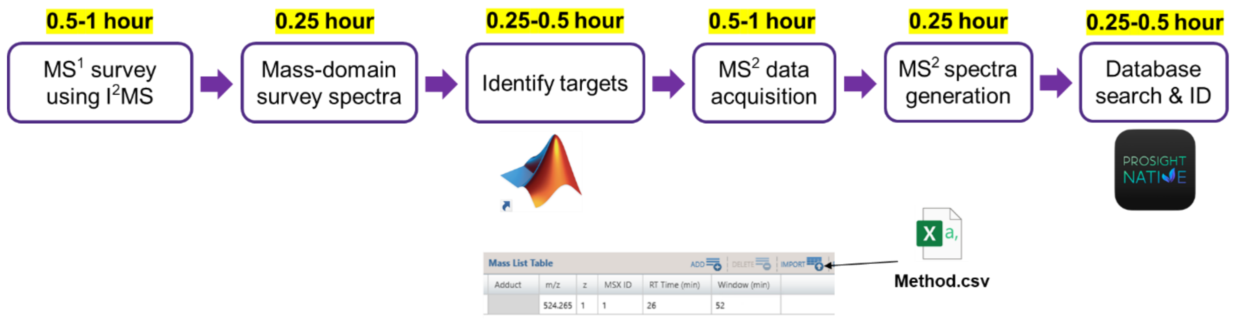

Supplementary Figure 16. Schematic AutoPiMS workflow and typical time in each step. the third step ("identify targets"), a Method.csv file is generated, which is subsequently imported into a method file (XCalibur 3.0 user interface on Orbitrap Exploris 480 is used as an example in the screenshot).

Supplementary References:

1. Hunt, A.L. et al. Extensive three-dimensional intratumor proteomic heterogeneity revealed by multiregion sampling in high-grade serous ovarian tumor specimens. *iScience* **24**, 102757 (2021).
2. Yin, R., Burnum-Johnson, K.E., Sun, X., Dey, S.K. & Laskin, J. High spatial resolution imaging of biological tissues using nanospray desorption electrospray ionization mass spectrometry. *Nat. Protoc.* **14**, 3445-3470 (2019).
