# Supplementary material for: Automated imaging and identification of proteoforms directly from ovarian cancer tissue
Source: Nat Commun. 2023 Oct 14;14:6478. doi: 10.1038/s41467-023-42208-3 (PMC10576781; doi:10.1038/s41467-023-42208-3)
Supplement: Supplementary file 4 — Description of Additional Supplementary Files [file 41467_2023_42208_MOESM4_ESM.pdf]

## Description of Additional Supplementary Files

**Supplementary Data 1**
**Description:**
Supplementary Table 1. List of 113 proteoform masses, ion counts, and relative abundances found in the survey line scan.
Supplementary Table 2. 87 proteoforms with unique m/z isolation windows and their signal/co-isolated signal.
Supplementary Table 3. AutoPiMS master table of MS2 in the I2MS mode (25 proteoforms).
Supplementary Table 4. AutoPiMS master table of MS2 in the I2MS mode (26 proteoforms, technical replicate 1).
Supplementary Table 5. AutoPiMS master table of MS2 in the I2MS mode (20 proteoforms, technical replicate 2).
Supplementary Table 6. AutoPiMS master table of MS2 in the ensemble mode (79 proteoforms). P-values were adjusted using Benjamini-Hochberg procedure at 1% FDR.
Supplementary Table 7. AutoPiMS master table of MS2 in the ensemble mode (112 proteoforms, technical replicate 1). P-values were adjusted using Benjamini-Hochberg procedure at 1% FDR.
Supplementary Table 8. AutoPiMS master table of MS2 in the ensemble mode (134 proteoforms, technical replicate 2). P-values were adjusted using Benjamini-Hochberg procedure at 1% FDR.
Supplementary Table 9. Master table of all 73 MS2-identified proteoforms.
Supplementary Table 10. 552 significant proteoform features in label-free quantitation. Q-values (1% FDR-adjusted p-values) were obtained using Benjamini-Hochberg procedure.
Supplementary Table 11. 597 significant proteoform features in label-free quantitation (technical replicate 1). Q-values (1% FDR-adjusted p-values) were obtained using Benjamini-Hochberg procedure.
Supplementary Table 12. 616 significant proteoform features in label-free quantitation (technical replicate 2). Q-values (1% FDR-adjusted p-values) were obtained using Benjamini-Hochberg procedure.
Supplementary Table 13. 303 proteoform features with significant differential ion counts in tumor and stroma in label-free quantitation and their corresponding intact mass tag database annotations. Q-values (1% FDR-adjusted p-values) were obtained using Benjamini-Hochberg procedure.
Supplementary Table 14. 618 proteoforms detected in the imaging dataset.
Supplementary Table 15. Master table of 17 proteoform signatures shown in Fig. 2.

**Supplementary Data 2**
**Description:** TDValidator output files for the MS/MS data presented in Figure 1.

**Supplementary Data 3**
**Description:** AutoPiMS code to recreate minimum dataset.
