# Supplementary material for: Automated imaging and identification of proteoforms directly from ovarian cancer tissue
Source: Nat Commun. 2023 Oct 14;14:6478. doi: 10.1038/s41467-023-42208-3 (PMC10576781; doi:10.1038/s41467-023-42208-3)
Supplement: Supplementary file 5 — Supplementary Data 1 [file 41467_2023_42208_MOESM5_ESM.zip › Supplementary Data 1/Updated list of SI tables/Supplementary table legends.docx]

**Supplementary Data 1**

**Table Legends for:**

**Automated imaging and identification of proteoforms directly from ovarian cancer tissue**

Supplementary Table 1. List of 113 proteoform masses, ion counts, and relative abundances found in the survey line scan.

Supplementary Table 2. 87 proteoforms with unique *m/z* isolation windows and their signal/co-isolated signal.

Supplementary Table 3. AutoPiMS master table of MS^2^ in the I^2^MS mode (25 proteoforms).

Supplementary Table 4. AutoPiMS master table of MS^2^ in the I^2^MS mode (26 proteoforms, technical replicate 1).

Supplementary Table 5. AutoPiMS master table of MS^2^ in the I^2^MS mode (20 proteoforms, technical replicate 2).

Supplementary Table 6. AutoPiMS master table of MS^2^ in the ensemble mode (79 proteoforms). P-values were adjusted using Benjamini-Hochberg procedure at 1% FDR.

Supplementary Table 7. AutoPiMS master table of MS^2^ in the ensemble mode (112 proteoforms, technical replicate 1). P-values were adjusted using Benjamini-Hochberg procedure at 1% FDR.

Supplementary Table 8. AutoPiMS master table of MS^2^ in the ensemble mode (134 proteoforms, technical replicate 2). P-values were adjusted using Benjamini-Hochberg procedure at 1% FDR.

Supplementary Table 9. Master table of all 73 MS^2^-identified proteoforms.

Supplementary Table 10. 552 significant proteoform features in label-free quantitation. Q-values (1% FDR-adjusted p-values) were obtained using Benjamini-Hochberg procedure.

Supplementary Table 11. 597 significant proteoform features in label-free quantitation (technical replicate 1). Q-values (1% FDR-adjusted p-values) were obtained using Benjamini-Hochberg procedure.

Supplementary Table 12. 616 significant proteoform features in label-free quantitation (technical replicate 2). Q-values (1% FDR-adjusted p-values) were obtained using Benjamini-Hochberg procedure.

Supplementary Table 13. 303 proteoform features with significant differential ion counts in tumor and stroma in label-free quantitation and their corresponding intact mass tag database annotations. Q-values (1% FDR-adjusted p-values) were obtained using Benjamini-Hochberg procedure.

Supplementary Table 14. 618 proteoforms detected in the imaging dataset.

Supplementary Table 15. Master table of 17 proteoform signatures shown in Fig. 2.
