# Supplementary material for: Automated imaging and identification of proteoforms directly from ovarian cancer tissue
Source: Nat Commun. 2023 Oct 14;14:6478. doi: 10.1038/s41467-023-42208-3 (PMC10576781; doi:10.1038/s41467-023-42208-3)
Supplement: Supplementary file 7 — Supplementary Data 3 [file 41467_2023_42208_MOESM7_ESM.zip › FisherPackage/Package/fisher-walkthrough.pdf]

Fisher Program Tutorial

Provided Example: Pierce Mix

	Protein Name	Protein Accession	Theo. Average Mass (Da)	Theo. Mono Mass (Da)
A	Human IGF-I LR3	P05019 (40-118)	9111.47	9105.34872
B	Human Thioredoxin	Q99757(60-166)	11865.52	11858.04393
C	Streptococcus dysgalactiae Protein G	P06654(223-413)	21442.61	21429.75915
D	Bovine Carbonic Anhydrase II	P00921	28981.29	28963.6881
E	Streptococcus Protein AG (chimeric)	P02976, P19909	50459.74	50429.84641
F	Escherichia coli Exo Klenow	P00582(324-928)	68001.15	67959.42515

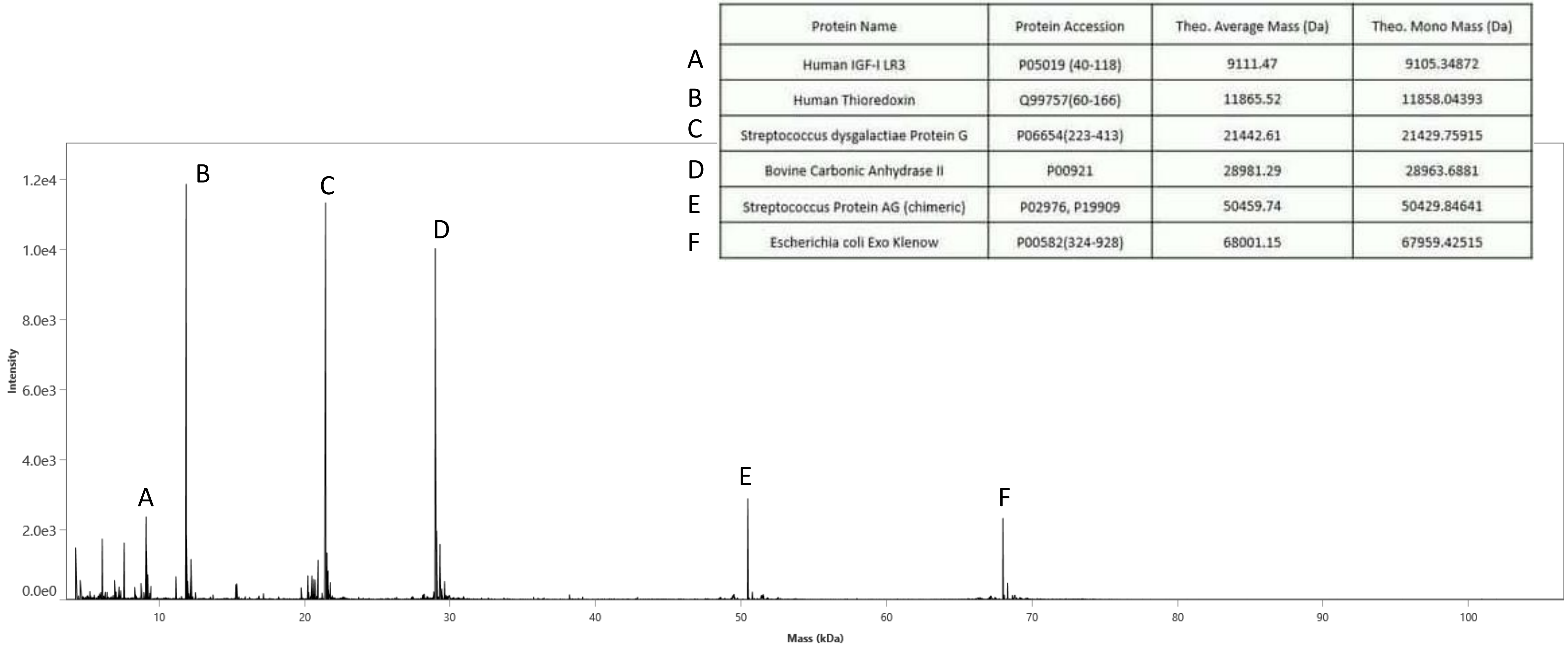

Required Input (in the same directory)

- Ion-table.csv
  - Derived from a *.dmt or *.i2ms file
- I2MSFisher.exe

Double-click on I2MSFisher.exe, and the output files should appear in the same directory.

Output

- A *.txt Target Report
  - Organized by target mass, target m/z (with ion count), and coisolates (with ion counts)
- A *.csv Target Report
  - Organized similarly to the *.txt Target Report, but with a consolidated coisolate count

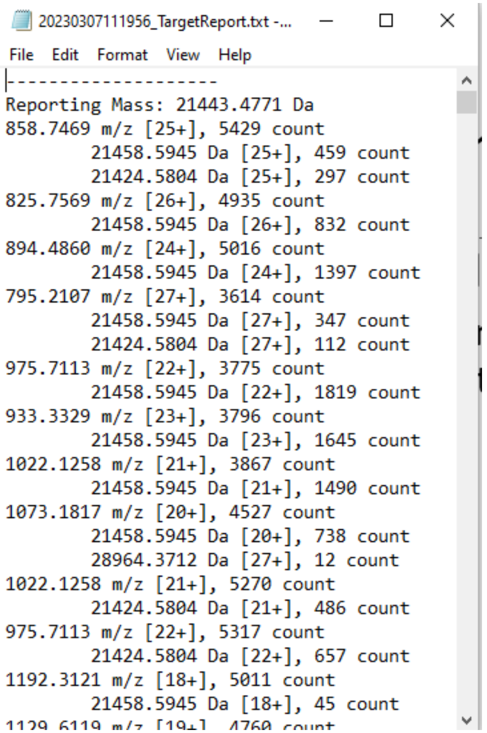


```
--------------------
Reporting Mass: 21443.4771 Da
858.7469 m/z [25+], 5429 count
        21458.5945 Da [25+], 459 count
        21424.5804 Da [25+], 297 count
825.7569 m/z [26+], 4935 count
        21458.5945 Da [26+], 832 count
894.4860 m/z [24+], 5016 count
        21458.5945 Da [24+], 1397 count
795.2107 m/z [27+], 3614 count
        21458.5945 Da [27+], 347 count
        21424.5804 Da [27+], 112 count
975.7113 m/z [22+], 3775 count
        21458.5945 Da [22+], 1819 count
933.3329 m/z [23+], 3796 count
        21458.5945 Da [23+], 1645 count
1022.1258 m/z [21+], 3867 count
        21458.5945 Da [21+], 1490 count
1073.1817 m/z [20+], 4527 count
        21458.5945 Da [20+], 738 count
        28964.3712 Da [27+], 12 count
1022.1258 m/z [21+], 5270 count
        21424.5804 Da [21+], 486 count
975.7113 m/z [22+], 5317 count
        21424.5804 Da [22+], 657 count
1192.3121 m/z [18+], 5011 count
        21458.5945 Da [18+], 45 count
1129.6119 m/z [19+], 4760 count
```

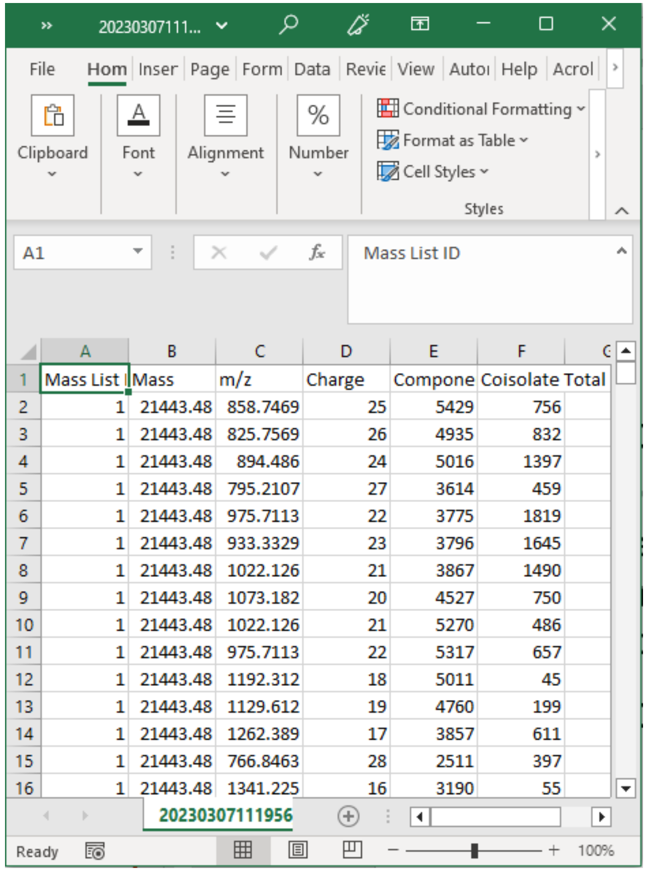

	A	B	C	D	E	F	G
1	Mass List I	Mass	m/z	Charge	Compone	Coisolate Total	
2	1	21443.48	858.7469	25	5429	756	
3	1	21443.48	825.7569	26	4935	832	
4	1	21443.48	894.486	24	5016	1397	
5	1	21443.48	795.2107	27	3614	459	
6	1	21443.48	975.7113	22	3775	1819	
7	1	21443.48	933.3329	23	3796	1645	
8	1	21443.48	1022.126	21	3867	1490	
9	1	21443.48	1073.182	20	4527	750	
10	1	21443.48	1022.126	21	5270	486	
11	1	21443.48	975.7113	22	5317	657	
12	1	21443.48	1192.312	18	5011	45	
13	1	21443.48	1129.612	19	4760	199	
14	1	21443.48	1262.389	17	3857	611	
15	1	21443.48	766.8463	28	2511	397	
16	1	21443.48	1341.225	16	3190	55	
